# Redefining high risk multiple myeloma with an APOBEC/Inflammation-based classifier

**DOI:** 10.1038/s41375-024-02210-0

**Published:** 2024-03-09

**Authors:** Sarah Grasedieck, Afsaneh Panahi, Matthew C. Jarvis, Faezeh Borzooee, Reuben S. Harris, Mani Larijani, Hervé Avet-Loiseau, Mehmet Samur, Nikhil Munshi, Kevin Song, Arefeh Rouhi, Florian Kuchenbauer

**Affiliations:** 1 Department of Microbiology and Immunology, University of British Columbia, 2125 East Mall, Vancouver, BC Canada; 2 Terry Fox Laboratory, BC Cancer Research Institute, Vancouver, BC Canada; 3 Department of Medicine, University of British Columbia, Vancouver, BC Canada; 4 Department of Biochemistry, Molecular Biology and Biophysics, University of Minnesota, Minneapolis, NC USA; 5 Department of Molecular Biology and Biochemistry, Faculty of Science, Simon Fraser University, Burnaby, BC Canada; 6 Department of Biochemistry and Structural Biology, University of Texas Health San Antonio, San Antonio, TX USA; 7 Howard Hughes Medical Institute, University of Texas Health San Antonio, San Antonio, TX USA; 8 IUCT-Oncopole Toulouse, Toulouse, France; 9 Dana-Farber Cancer Institute, Harvard Medical School, Boston, MA USA; 10 Leukemia/Bone Marrow Transplant Program of British Columbia, Vancouver General Hospital, BC Cancer, Vancouver, BC Canada

**Keywords:** Myeloma, Cancer genomics, Translational research

## Abstract

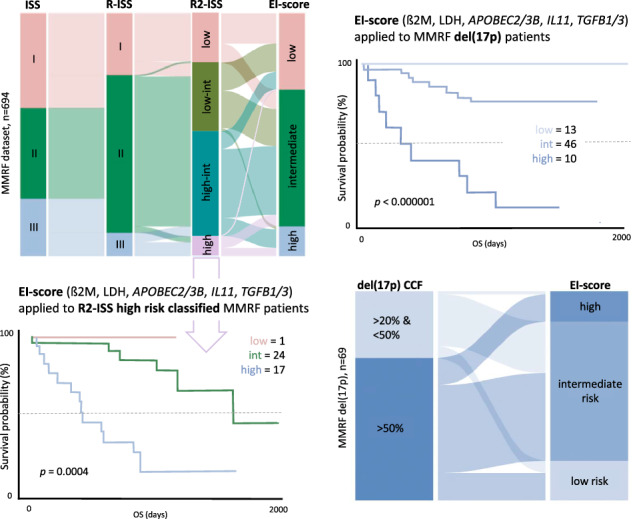

## To the Editor:

Recent studies have identified mutational genomic signatures introduced by *Apolipoprotein B mRNA-Editing Catalytic Polypeptide-like* (APOBEC) deaminases as well as inflammatory processes as being pivotal for MM onset and progression [1–4]. Although these recent insights provide a better understanding of MM pathogenesis, they have not yet been translated into clinical applications such as MM risk stratification. The current standards for MM patient risk classification are the International Staging System (ISS), the Revised ISS (R-ISS) and the second revision of the R-ISS (R2-ISS) introduced between 2005 and 2022, respectively [5]. All scores are based on clinical parameters reflecting tumor burden, and the newer R-ISS and R2-ISS further incorporate high-risk cytogenetics [5]. Considering that most risk-defining chromosomal abnormalities reflect early events in MM cells [6], we concluded that tumor burden and/or cytogenetics-based classifiers might not accurately reflect the dynamics of disease progression in MM patients. Therefore, we hypothesized that a predictive score which reflects molecular mechanisms that drive MM progression, can improve the accuracy of current MM risk classifiers. To test this hypothesis, we constructed and validated a proof-of-principle risk classifier called Editor/Inflammation- or EI-score, which combines mRNA levels of survival-associated *APOBEC* genes, pro/anti-inflammatory genes as well as clinical markers for MM disease burden.

Data from 1143 patients with newly diagnosed MM (NDMM) and available survival information was obtained through the CoMMpass database version IA14, which was generated as part of the Multiple Myeloma Research Foundation (MMRF) Personalized Medicine Initiatives (www.themmrf.org). ISS, R-ISS and R2-ISS staging information was available for 1113, 694, and 694 patients, respectively. For 599 patients, information on both blood parameters and RNA-seq was available. As an independent validation cohort, we analyzed clinical, cytogenetic, and RNA-seq data from 263 NDMM patients treated as part of the IFM/DFCI 2009 trial (ClinicalTrials.gov identifier: NCT01191060) [7]. IFM/DFCI patients were treated with Bortezomib, Lenalidomide and Dexamethasone (VRD) alone or with VRD+autologous stem cell transplantation (ASCT). All patient baseline characteristics (CoMMpass and IFM/DFCI) are summarized in Table S1. A stepwise workflow for the evaluation and selection of individual features and multivariate models in the MMRF CoMMpass dataset is shown in Fig. S1 and described in detail in the Supplementary Methods.

To translate recent whole genome- and RNA-sequencing findings into a predictive score, we pre-selected 163 features, including demographic, clinical, genomic, and cytogenetic information, as well as inflammatory signaling and nucleotide editing-associated mRNA covariates from the MMRF CoMMpass dataset (Fig. S1). Of the 163 tested variables, 25 for overall survival (OS) and 21 for progression-free survival (PFS) showed significant time-to-event outcomes. Notably, only one out of five cytogenetic features, namely +1q/amp1q (Fig. S2), passed our stringent selection criteria in 599 NDMM patients. In line with our hypothesis, we found that mRNA levels of individual *APOBEC* genes as well as APOBEC-induced genomic mutational signatures (calculated in form of both COSMIC single-base substitution (*SBS*) signature and APOBEC mutation enrichment score [8, 9]) were associated with inferior OS and PFS (Fig. S2). As the rationale of this study was not to provide a score for immediate clinical application but rather to determine if combining APOBEC and inflammation-associated gene expression variables holds prognostic merit for MM patients, we reduced our feature set to only the most significant variables that were associated with both OS and PFS. We then combined all age- and treatment-independent prognostic variables that passed our selection criteria (and for RNA parameters, showed a median expression >5 fragments per kilobase per million) into multivariate CoxPH models, excluding patient cytogenetics and mutational signatures. This included the following parameters: ß2M, Creatinine, Hemoglobin, LDH, *APOBEC2, APOBEC3A, APOBEC3B, APOBEC3C, APOBEC3D, APOBEC3F, APOBEC3G, IL10, IL11, IL17C, IL27, IFNG, TGFB1, TGFB3, IL22RA1, IL2RA, TGFBR3, CXCL13*. Patient age >75 y was excluded due to the inclusion criteria of the IFM/DFCI2009 study (18–65 y). The multivariate model with the highest predictive performance while retaining as few parameters as possible included the following seven features: ß2M, LDH, *APOBEC2*, *APOBEC3B*, *IL11*, *TGFB1*, *TGFB3*. Based on these seven parameters, we devised a streamlined scoring formula that relies on maximally selected rank statistics established cut-offs and incorporates weights derived from the rounded integer multivariate CoxPH *z*-score of each parameter. Although we detected strong correlation among expression levels of most members of the APOBEC family, there was no significant positive correlation between *APOBEC2* and *APOBEC3B* (Pearson’s *R* = 0.039), which are both part of the EI-score (Fig. S3). The distribution of each expressed EI-score gene in the different MMRF CoMMpass cytogenetic and age groups is shown in Fig. S4.

**Fig. 1:**
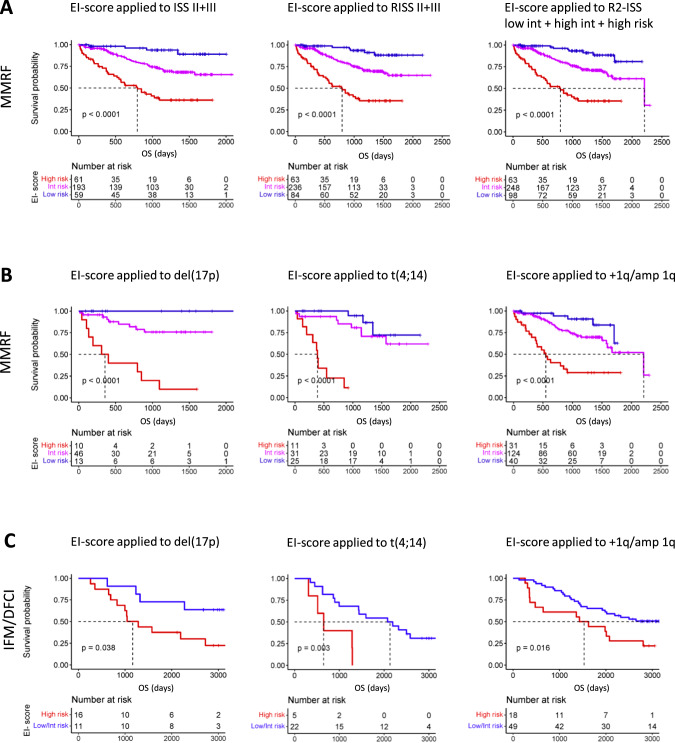
The EI-score reclassifies MM patients and identifies novel prognostic MM subgroups. Shown are graphical representations of OS Kaplan–Meier estimates based on the application of the EI-score[OS] to (**A**) MMRF CoMMpass patients who were stratified into ISS and R-ISS stage II and III as well as into R2-ISS low intermediate, high intermediate, and high risk groups. **B** MMRF CoMMpass patients carrying del(17p), t(4;14), or +1q, and (**C**) IFM/DFCI patients carrying del(17p), t(4;14), or +1q reclassified by the EI-score.

To evaluate the prognostic accuracy of the EI-score compared to ISS, R-ISS, R2ISS, and mSMART_cyto_ (a reduced version of the Mayo clinic mSMART score: https://www.msmart.org, based on the presence of t(4;14), t(14;16), t(14;20), +1q and/or del(17p)), we computed performance metrics for the outcome prediction of each score in MMRF CoMMpass patients (Table 1, Fig. S5). The EI-score achieved the best performance for OS and PFS prediction (*n* = 599; Concordance index (C_i_) 0.7 and 0.69, respectively), followed by R2-ISS (*n* = 694; C_i_ 0.66 and 0.61), ISS (*n* = 1113; C_i_ 0.66 and 0.6), R-ISS (*n* = 690; C_i_ 0.64 and 0.6), and mSMART_cyto_ (*n* = 823; C_i_ 0.58 and 0.54). We then successfully validated the EI-score in the IFM/DFCI2009 NDMM cohort (*n* = 263) (Fig. S5), representing a homogeneously treated patient collective. Notably, addition of EI-score gene expression information to ISS, R-ISS, R2-ISS (Fig. 1A, Table 1, Table S2), and mSMART_cyto_, improved the performance of each classifier significantly. Moreover, applying the EI-score exclusively to MM patient subgroups with del(17p), +1q, and t(4;14) allowed to identify previously unrecognized favorable risk patients with adverse risk cytogenetics in the MMRF CoMMpass (Fig. 1B, Fig. S6) as well as in the IFM/DFCI cohort (Fig. 1C). In line, we found that del(17p), +1q, and t(4;14) patients with a *high* EI-score, displayed an enrichment of APOBEC-induced genomic mutations compared to *low*/*intermediate* EI-score patients (Fig. S7). These results demonstrate that the integration of APOBEC and inflammatory cytokine mRNA levels improve the prognostic capacity of chromosomal abnormalities, which are currently viewed as risk class defining. To adjust for the heterogeneous treatment protocols of patients included in the MMRF CoMMpass dataset, we also conducted a sub-analysis of MM patients receiving Cyclophosphamide, Bortezomib, Dexamethasone (CyBorD) or VRD ± ASCT (Fig. S8) and a sub-analysis of MM patients receiving VRD ± ASCT + maintenance therapy (Fig. S9), in which the EI-score also outperformed ISS, R-ISS, and R2-ISS. A possible explanation why APOBEC family members have so far not been part of probe-based mRNA classifiers such as EMC-92 [10] and UAMS-70 [11] is likely due to their high sequence similarity resulting in probe cross-hybridization and multimapping to several APOBEC members [12]. The high hazard ratio and predictive performance of *APOBEC3B* expression for adverse PFS and OS which appears to be independent from that of APOBEC-induced mutational signatures, likely reflects APOBEC3B’s additional involvement in MM pathogenesis through immune editing, viral and retroelement restriction, DNA demethylation, and tissue homeostasis [13]. Although APOBEC3B-induced C-to-U lesions are typically resolved by DNA repair response mechanisms, they can promote chronic replication stress and thus contribute to MM development, which could be a reason for the high predictive value we observed for APOBEC mRNA levels with MM patient outcomes. The MM microenvironment is characterized by a desynchronized cytokine milieu, with imbalanced pro- and anti-inflammatory factors that impact on MM and niche cells. Besides their general role in inflammatory processes, IL-11 as well as TGF-ß have both been implicated in the growth and differentiation block of osteoblasts [14], which in turn modulates MM cell activity. Likewise, *APOBEC3B* and *APOBEC2* upregulation has been linked to systemic inflammation [13], suggesting that a pro-inflammatory microenvironment in MM cells could drive *APOBEC2* and *APOBEC3B* expression. However, the precise regulation and function of APOBEC2 and APOBEC3B in MM cells still needs to be defined.

**Table 1:** Incorporation of EI-score gene expression information improves the performance of established risk classifiers in the MMRF CoMMpass dataset.

Multivariate model	Cox Proportional Hazard Regression				ML Model ROC-AUC			*n*	
Progression free survival (PFS)	Likeli-hood ratio	Wald test	Log rank test	Concor-dance index (C_i_)	Random forest	gradient boosting	Negative binomial	total	events
(1) mSMARTcyto	10.47	11.02	11.04	0.54	0.48	0.52	0.52	817	391
mSMARTcyto + *APOBEC2*, *APOBEC3B*	49.15	58.82	62.15	0.60	0.54	0.57	0.58	645	316
mSMARTcyto + *IL11, TGFB1, TGFB3*	24.19	24.64	24.87	0.58	0.55	0.56	0.57	645	316
mSMARTcyto + APOBECs + Cytokines	56.46	60.62	62.24	0.63	0.6	0.6	0.61	645	316
(2) ISS	59.41	59.28	60.75	0.60	0.6	0.6	0.59	1113	573
ISS + *APOBEC2, APOBEC3B*	79.16	87.18	90.55	0.63	0.62	0.63	0.63	746	375
ISS + *IL11, TGFB1, TGFB3*	52.04	51.97	52.98	0.62	0.61	0.62	0.61	746	375
ISS + APOBECs + Cytokines	96.22	103.5	107.7	0.65	0.62	0.65	0.65	746	375
(3) R-ISS	36.92	36.91	37.05	0.60	0.55	0.55	0.54	690	319
R-ISS + *APOBEC2, APOBEC3B*	71.75	79.01	83.4	0.65	0.6	0.61	0.6	536	254
R-ISS + *IL11, TGFB1, TGFB3*	52.84	52.14	53.06	0.65	0.56	0.59	0.6	536	254
R-ISS + APOBECs + Cytokines	90.28	96.5	102.4	0.68	0.61	0.62	0.63	536	254
(4) R-ISS-nocyto	38.58	38	38.46	0.60	0.58	0.59	0.59	526	292
R-ISS-nocyto + *APOBEC2, APOBEC3B*	77.63	85.04	90.72	0.66	0.65	0.67	0.67	419	223
R-ISS-nocyto + *IL11, TGFB1, TGFB3*	56.02	54.22	55.46	0.66	0.62	0.65	0.66	419	223
R-ISS-nocyto + APOBECs + Cytokines	92.5	97.33	104.8	0.69	0.66	0.69	0.68	419	223
(5) R2-ISS	38.17	39.37	39.83	0.61	0.57	0.65	0.64	694	318
R2-ISS + *APOBEC2, APOBEC3B*	77.12	85.83	90.16	0.65	0.67	0.69	0.69	543	255
R2-ISS + *IL11, TGFB1, TGFB3*	54.51	54.64	55.87	0.64	0.63	0.7	0.69	543	255
R2-ISS + APOBECs + Cytokines	94.25	100.2	106.5	0.68	0.68	0.72	0.73	543	255
(6) blood parameters (ß2M, LDH)	38.6	43.57	44.36	0.62	0.61	0.6	0.6	872	420
ß2M + LDH + *APOBEC2, APOBEC3B*	97.3	103	108.4	0.67	0.63	0.65	0.66	599	285
ß2M + LDH + *IL11, TGFB1, TGFB3*	70.43	69.52	71.11	0.66	0.63	0.64	0.65	599	285
ß2M/LDH + APOBECs + Cytokines (EI-score)	**114.4**	**120.1**	**126.4**	**0.69**	**0.65**	**0.67**	**0.66**	599	285
7) gene expression only:	-	-	-	-	-	-	-	-	-
*APOBEC2, APOBEC3B*	45.01	53.23	55.81	0.58	0.56	0.56	0.54	767	390
*IL11, TGFB1, TGFB3*	18.86	19.19	19.43	0.57	0.53	0.55	0.55	767	390
APOBECs + Cytokines	63.51	71.75	74.84	0.62	0.6	0.6	0.6	767	390

Overall survival (OS)	Likeli-hood ratio	Wald test	Log rank test	Concor-dance index (C_i_)	Random forest	gradient boosting	Negative binomial	total	events
(1) mSMARTcyto	18.46	20.22	20.35	0.58	0.52	0.55	0.56	817	173
mSMARTcyto + *APOBEC2*, *APOBEC3B*	47.67	53.18	56.15	0.66	0.63	0.64	0.64	645	141
mSMARTcyto + *IL11, TGFB1, TGFB3*	52.42	51.74	54.5	0.66	0.6	0.63	0.65	645	141
mSMARTcyto + APOBECs + Cytokines	82.76	82.65	88.22	0.71	0.66	0.69	0.69	645	141
(2) ISS	75.21	71.09	76.12	0.66	0.54	0.65	0.63	1113	266
ISS + *APOBEC2, APOBEC3B*	92.07	94.68	102	0.72	0.65	0.7	0.7	746	172
ISS + *IL11, TGFB1, TGFB3*	77.62	74.89	79.61	0.69	0.63	0.69	0.69	746	172
ISS + APOBECs + Cytokines	121.5	125.2	132.8	0.74	0.68	0.73	0.73	746	172
(3) R-ISS	39.14	39.11	39.23	0.64	0.55	0.61	0.6	690	142
R-ISS + *APOBEC2, APOBEC3B*	64.36	65.24	69.48	0.71	0.62	0.67	0.68	536	116
R-ISS + *IL11, TGFB1, TGFB3*	62.57	60.36	64.59	0.70	0.6	0.68	0.67	536	116
R-ISS + APOBECs + Cytokines	93.43	90.28	99.19	0.74	0.66	0.71	0.7	536	116
(4) R-ISS-nocyto	33.15	32.43	32.63	0.64	0.52	0.63	0.61	526	127
R-ISS-nocyto + *APOBEC2, APOBEC3B*	55.98	58.99	62.18	0.72	0.65	0.69	0.68	419	105
R-ISS-nocyto + *IL11, TGFB1, TGFB3*	57.76	55.47	59.1	0.71	0.65	0.7	0.7	419	105
R-ISS-nocyto + APOBECs + Cytokines	83.75	83.38	89.97	0.74	0.68	0.73	0.73	419	105
(5) R2-ISS	47.16	48.76	49.99	0.67	0.57	0.65	0.64	694	142
R2-ISS + *APOBEC2, APOBEC3B*	72.25	75.82	80.15	0.72	0.67	0.69	0.69	543	116
R2-ISS + *IL11, TGFB1, TGFB3*	67.04	65.98	69.72	0.71	0.63	0.7	0.69	543	116
R2-ISS + APOBECs + Cytokines	96.89	97.7	104.5	0.74	0.68	0.72	0.73	543	116
(6) blood parameters (ß2M, LDH)	58.47	54.58	58.09	0.67	0.64	0.67	0.66	872	184
ß2M + LDH + *APOBEC2, APOBEC3B*	92.73	93.2	102.6	0.74	0.69	0.72	0.72	599	127
ß2M + LDH + *IL11, TGFB1, TGFB3*	81.34	78.4	83.48	0.73	0.67	0.72	0.7	599	127
ß2M/LDH + APOBECs + Cytokines (EI-score)	**119.3**	**119.1**	**130.9**	**0.76**	**0.72**	**0.74**	**0.75**	599	127
(7) gene expression only:	-	-	-	-	-	-	-	-	-
*APOBEC2, APOBEC3B*	48.03	53.18	57	0.64	0.54	0.61	0.61	767	178
*IL11, TGFB1, TGFB3*	35.03	36.34	37.99	0.63	0.55	0.61	0.59	767	178
APOBECs + Cytokines	85.14	87.42	93.32	0.69	0.64	0.67	0.67	767	178

The bold values highlight the performance metrics achieved by our developed EI-score.

In this study, we have developed the EI-score which serves as an important proof-of-concept, demonstrating that inclusion of molecular markers that reflect disease progression can improve MM risk assessment. Although our data highlights the limitations of cytogenetics-based risk stratifiers, ISS, R-ISS and R2-ISS represent the current clinical standard due to their accessibility. Eventually, the development of more contemporary stratification systems will be necessary to improve risk- and treatment stratifications of MM patients.

### Supplementary information

Supplementary Material

## Data Availability

MMRF sequencing data is available through the CoMMpass database version IA14 (www.themmrf.org). DFM/DFCI 2009 sequencing data can be requested through Nikhil_munshi@dfci.harvard.edu.
