# Supplementary material for: Redefining high risk multiple myeloma with an APOBEC/Inflammation-based classifier
Source: Leukemia. 2024 Mar 9;38(5):1172–7. doi: 10.1038/s41375-024-02210-0 (PMC11073955; doi:10.1038/s41375-024-02210-0)
Supplement: Supplementary file 1 — Supplementary Material [file 41375_2024_2210_MOESM1_ESM.pdf]

Supplementary Materials and Methods

Patient cohorts and available data

Data from 1,143 patients with newly diagnosed MM (NDMM) and available survival information was obtained through the CoMMpass database version IA14, which was generated as part of the Multiple Myeloma Research Foundation Personalized Medicine Initiatives (www.themmrf.org). ISS, R-ISS and R2-ISS staging information was available for 1113, 694 and 694 patients, respectively. For 599 patients, information on both blood parameters and RNA-seq from CD138+ sorted bone marrow (BM) cells was available. As an independent validation cohort, we utilized data collected from 263 NDMM patients in the IFM/DFCI 2009 clinical trial (ClinicalTrials.gov identifier: NCT01191060) with available clinical, cytogenetic (FISH) (del(17p), t(4;14), t(14;16)) and RNA-seq data from CD138+ BM cells. IFM/DFCI MM patients were treated with bortezomib, lenalidomide and dexamethasone (VRD) alone or with VRD+autologous stem cell transplantation (ASCT). All patient baseline characteristics (MMRF CoMMpass and IFM/DFCI) are summarized in **Table S1.**

Feature selection, model building, performance evaluation, and validation

A stepwise workflow for the evaluation and selection of individual features and multivariate models in the MMRF CoMMpass data is shown in **Figure S1**. Transcript per million (TPM) normalized gene expression and canonical genomic mutational variants per patient were obtained through the MMRF CoMMpass database. First, we performed univariate Cox Proportional Hazard (CoxPH) analyses using the R survival package1 on 163 variables comprised of demographic features (n=6), clinical blood parameters (n=19), cytogenetic abnormalities (n=5), cell surface receptors assessed by flow cytometry (n=9), mRNA transcript levels of APOBEC, pro/anti-inflammatory cytokines and cytokine receptors

(n=119), total mutational burden, as well as computed single base substitution (SBS) genomic mutational signatures linked to ageing and APOBEC activity (n=5). SBS mutational signatures as listed in COSMIC version 3 (SBS1/5 = age-associated, SBS2/13 = APOBEC-associated) as well as an improved C-to-T/Cto-G in 5'TC(A/T motifs) APOBEC enrichment score were calculated as described by Jarvis et al.[2]. Applying log likelihood or Chi-square tests, all of these 163 features were then assessed in multivariate CoxPH models to select only variables that showed additional impacted on the association with patient survival when combined with age and/or first-line treatment. All continuous parameters (including RNA transcript levels and blood measurement data) were transformed to categorical variables applying maximally selected rank statistics (R survival package) to define the cutoff between high and low patient groups for each individual feature. Although maximally selected rank statistics calculated cut-offs were used to calculate the final cut-offs in our presented models and scores, we also computed median- as well as 1st and 4th quartile cut-offs for all candidate variables. Only the most robust variables moved on to multivariate testing that were significantly associated with both PFS and OS in at least two of the four tested cut-off categories. All age- and treatment-independent prognostic variables that passed these criteria (and for RNA parameters, showed a median expression >5 fragments per kilobase per million) were then combined into multivariate CoxPH models, excluding patientcytogenetics and mutational signatures. This included the following parameters: ß2M, Creatinine, Hemoglobin, LDH, *APOBEC2, APOBEC3A, APOBEC3B, APOBEC3C, APOBEC3D, APOBEC3F, APOBEC3G, IL10, IL11, IL17C, IL27, IFNG, TGFB1, TGFB3, IL22RA1, IL2RA, TGFBR3, CXCL13*. Patient age >75y was excluded due to the inclusion criteria of the IFM/DFCI2009 study (18-65y). The multivariate model with the highest predictive performance based on these parameters retained the following seven features: ß2M, LDH, *APOBEC2, APOBEC3B, IL11, TGFB1, TGFB3*. Based on these seven parameters, we devised a streamlined scoring formula that relies on

maximally selected rank statistics to establish cut-offs and incorporates weights derived from the rounded integer multivariate CoxPH z-score of each parameter:

<u>EI-score[OS]:</u> (*APOBEC2*: ≤0.20=0, >0.20=3) + (*APOBEC3B*: ≤3.79=0, >3.79=3) + (*IL11*: ≤0.48=0, >0.48 =2.5) + (*TGFB1*: ≤0.11=0, >0.11 =1) + (*TGFB3*: ≤0.10=0, >0.10=2) + (ß2M: ≤4.22=0, >4.22=4) + (LDH: ≤3.18=0, >3.18=2)

<u>EI-score[PFS]:</u> (*APOBEC2*: ≤0.19=0, >0.19=4) + (*APOBEC3B*: ≤6.91=0, >6.91=3) + (*IL11*: ≤0.56=0, >0.56 =2) + (*TGFB1*: ≤0.43=0, >0.43 =1) + (*TGFB3*: ≤0.03=0, >0.03=1) + (ß2M: ≤3.33=0, >3.33=4) + (LDH: ≤3.08=0, >3.08=2)

Our rationale for calculating two separate scores with different weighting was informed by our observation that prediction of PFS in MMRF patients appeared to rely more heavily on low *APOBEC2* expression levels (PFS z=-4.34, p=0.00001 vs. OS z=-3.56, p=0.0004), and was improved by inclusion of the SBS1 ageing- and SBS2 APOBEC-associated mutational signatures (PFS: p=0.004 and p=0.005, respectively vs OS: not significant), whereas OS prediction in this patient cohort was improved by the inclusion of creatinine blood levels and RNA levels of inflammatory cytokines such as *TGFB3* and *IL11*. Prediction accuracy metrics of multivariate models were determined through multivariate CoxPH analyses and by training three individual machine-learning models after randomly splitting the data into training (75%) and testing (25%) cohorts applying the Monte Carlo cross-validation approach, including a random forest (rf), gradient boosting (gbm), and negative binomial (nb) model, both for overall survival (OS) and progression free survival (PFS) prediction (*R caret, tidymodels, tidyverse, ranger, MLeval*)[3-7]. Model performance on the testing data was evaluated based on receiver operating characteristic (ROC) as well as precision recall (PR) efficiency and recorded in the form of ROC area under the curve (AUC) values. Reproducibility was assessed using a leave-one-out approach for multivariate Cox regression, and 10-fold cross validation for all machine learning approaches. An independent validation in the IFM/DFCI cohort was performed by applying the score formula.

Stratification of patients into EI-score *high*, *intermediate*, and *low* groups was determined based on natural valleys in the score distribution. To adjust for the heterogeneous treatment protocols of patients included in the MMRF dataset, we also performed sub analyses for MM patients receiving CyBorD or VRD ± ASCT (**Figure S8**) and VRD+ASCT ±maintenance therapy (**Figures S9**).

List of Supplementary Figures and Tables

Figure S1. Feature selection and multivariate model construction workflow.

Figure S2. Univariate hazard ratios for survival-associated variables in the MMRF CoMMpass dataset. Values greater than 1 represent decreased survival.

Figure S3. Correlation of APOBEC expression in MMRF CoMMpass patients. **A.** Correlation between *APOBEC2* and *APOBEC3B* expression (FPKM). **B**. Pearson's correlation between transcript levels of all *APOBEC* genes in MMRF patients.

Figure S4. Expression in FPKM of each EI-score gene parameter in MMRF patients stratified by **A.** cytogenetics and **B.** age quartiles.

Figure S5. Graphical representation of EI-score Kaplan-Meier estimates in the MMRF CoMMpass and IFM/DFCI 2009 datasets. **A.** EI-score[OS] and **B.** EI-score[PFS] calculated for each patient in the MMRF CoMMpass dataset. Dichotomization into two risk groups was performed based on maximally selected rank statistics. **C.** EI-score[OS] and **D.** EI-score[PFS] calculated for each patient in the IFM/DFCI 2009 dataset. **E-F**. Graphical representation of Kaplan-Meier estimates based on EI-score[OS] and EI-score[PFS] calculated for each patient in the MMRF CoMMpass dataset. Stratification into *high*-, *intermediate*- and *low*-risk groups was performed based on naturally occurring valleys in the EI-score[OS] and EI-score[PFS] data distribution.

Figure S6. Application of EI-score to MMRF patients with **A.** chr1q gain stratified by1 copy gain (+(1q)) and gain ≥2 copies (amp(1q)), and **B.** del(17p) stratified by cancer clonal fraction (CCF).

Figure S7. Quantified enrichment of APOBEC-induced genomic mutations (SBS2, SBS13) in MMRF CoMMpass patients stratified by EI-score risk group (*low*, *intermediate*, *high*) in 3 cytogenetic patient subgroups: **A.** del(17p), **B.** t(4;14), and **C.** +1q.

Figure S8. Graphical presentations of Kaplan Meier analysis showing the application of 3-group EI-score risk classification of MMRF CoMMpass patients who **A.** received CyBorD as induction regimen with or without ASCT or **B.** received VRD as induction regimen with or without ASCT. ASCT = autologous stem cell transplant; CyBorD = Bortezomib plus cyclophosphamide and dexamethasone; VRD = Bortezomib (Velcade), lenalidomide (Revlimid), and "low dose" dexamethasone.

Figure S9. Graphical presentations of Kaplan Meier analyses considering only MMRF CoMMpass patients who received VRD+ASCT+maintenance treatment. **A.** shows patient risk stratification using a two-group EI-score (high/low), **B.** shows application of 3-group EI-score version (high/intermediate/low). PFS is shown on the left and OS on the right. **C.** shows OS prediction when ISS is applied, and **D**. depicts OS prediction when R-ISS is applied to the same group of patients. ASCT = autologous stem cell transplant; VRD = Bortezomib, lenalidomide, and "low dose" dexamethasone.

Table S1. Summary of patient baseline characteristics in MMRF and IFM/DFCI 2009 datasets.

Table S2. Incorporation of EI-score gene expression information improves the performance of established risk classifiers in the IFM/DFCI 2009 dataset.

Suppl. References

1. Therneau, T. A Package for Survival Analysis in S. version 2.38. (2015).
2. Jarvis MC, Ebrahimi D, Temiz NA, Harris RS. Mutation Signatures Including APOBEC in Cancer Cell Lines. *JNCI Cancer Spectr*. 2018;2(1):pky002.
3. Kuhn, M., Caret: Classification and Regression Training. R package version 6.0-86. 2020.
4. Kuhn M, W.H., Tidymodels: a collection of packages for modeling and machine learning using tidyverse principles. 2020.
5. Wickham H, A.M., Bryan J, Chang W, McGowan LD, François R, Grolemund G, Hayes A, Henry L, Hester J, Kuhn M, Pedersen TL, Miller E, Bache SM, Müller K, Ooms J, Robinson D, Seidel DP, Spinu V, Takahashi K, Vaughan D, Wilke C, Woo K, Yutani H, Welcome to the Tidyverse. Journal of Open Source Software, 4(43), 1686., 2019.
6. Wright, M.N. and A. Ziegler, ranger: A Fast Implementation of Random Forests for High Dimensional Data in C++ and R. Journal of Statistical Software, 2017. **77**(1): p. 1-17.
7. John, C.R., Machine Learning Model Evaluation Version 0.3. 2020.

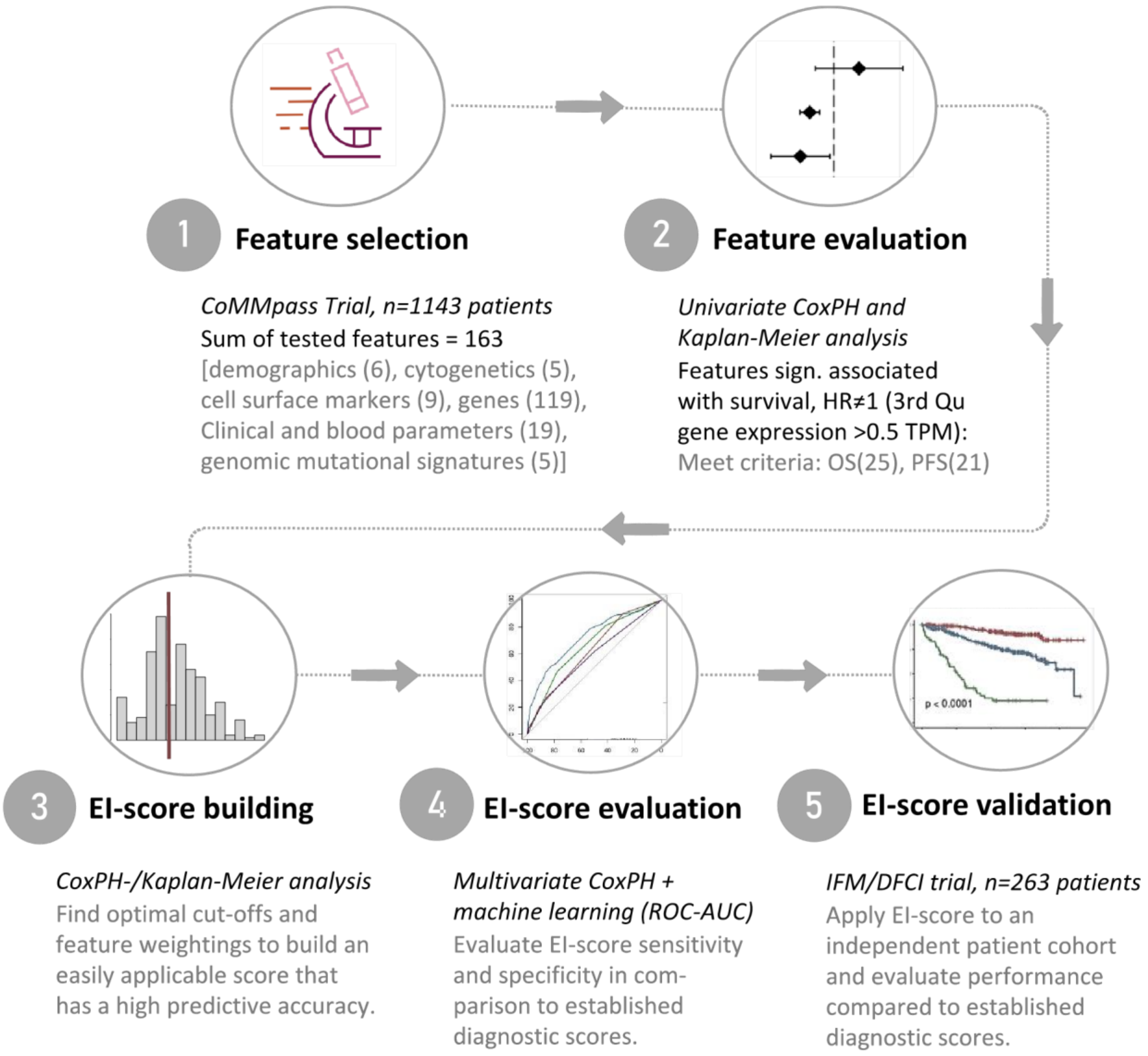
1
Feature selection
CoMMpass Trial, n=1143 patients
Sum of tested features = 163
[demographics (6), cytogenetics (5), cell surface markers (9), genes (119), Clinical and blood parameters (19), genomic mutational signatures (5)]
2
Feature evaluation
Univariate CoxPH and Kaplan-Meier analysis
Features sign. associated with survival, HR≠1 (3rd Qu gene expression >0.5 TPM):
Meet criteria: OS(25), PFS(21)
p < 0.0001
3
EI-score building
CoxPH-/Kaplan-Meier analysis
Find optimal cut-offs and feature weightings to build an easily applicable score that has a high predictive accuracy.
4
EI-score evaluation
Multivariate CoxPH + machine learning (ROC-AUC)
Evaluate EI-score sensitivity and specificity in comparison to established diagnostic scores.
5
EI-score validation
IFM/DFCI trial, n=263 patients
Apply EI-score to an independent patient cohort and evaluate performance compared to established diagnostic scores.

Figure S1.

Endpoint	Subgroup	n	HR	CI	p
PFS	t(4;14)	817	1.22	(0.93...1.61)	0.157
	t(14;16)		1.07	(0.66...1.74)	0.779
	t(14;20)		1.31	(0.58...2.94)	0.512
	Del(17p)		1.06	(0.79...1.44)	0.689
	+1q/gain1q		1.36	(1.11...1.66)	0.003
	TP53mut		1.51	(1.01...2.27)	0.046
	Total SNVs >93	728	0.86	(0.71...1.05)	0.138
	SBS1 (ageing), 4th Qu	931	0.87	(0.71...1.08)	0.220
	SBS5 (ageing), 4th Qu		1.04	(0.84...1.29)	0.715
	SBS40 (ageing), 4th Qu		1.27	(0.91...1.77)	0.159
	patient age >75	1143	2.09	(1.70...2.58)	<0.001
	SBS2 (APOBEC), 4th Qu	931	1.40	(1.08...1.82)	0.010
	SBS13 (APOBEC), 4th Qu		1.46	(1.05...2.03)	0.026
	APOBEC enr. score 4th Qu	726	1.42	(1.12...1.79)	0.004
	APOBEC2 >0.2	767	1.75	(1.37...2.24)	<0.001
	APOBEC3A >0.39		1.52	(1.23...1.88)	<0.001
	APOBEC3B >6.9		2.32	(1.76...3.07)	<0.001
	APOBEC3C >14.5		1.76	(1.42...2.18)	<0.001
	APOBEC3D >5.1		1.68	(1.26...2.23)	<0.001
	APOBEC3F >5.75		1.50	(1.13...1.99)	0.005
	APOBEC3G >23.2		1.49	(1.14...1.95)	0.003
	APOBEC3H >0.96		1.43	(1.14...1.81)	0.002

Endpoint	Subgroup	n	HR	CI	p
OS	t(4;14)	817	1.30	(0.87...1.93)	0.204
	t(14;16)		0.88	(0.39...2.00)	0.767
	t(14;20)		2.63	(1.08...6.41)	0.033
	Del(17p)		1.41	(0.93...2.13)	0.110
	+1q/gain1q		1.80	(1.34...2.43)	<0.001
	TP53mut		1.87	(1.08...3.22)	0.025
	Total SNVs >93	728	1.38	(0.94...2.03)	0.104
	SBS1 (ageing), 4th Qu	931	0.89	(0.65...1.23)	0.497
	SBS5 (ageing), 4th Qu		0.97	(0.70...1.34)	0.847
	SBS40 (ageing), 4th Qu		1.48	(0.94...2.33)	0.088
	patient age >75	1143	2.51	(1.90...3.32)	<0.001
	SBS2 (APOBEC), 4th Qu	931	1.52	(1.06...2.19)	0.024
	SBS13 (APOBEC), 4th Qu		2.17	(1.43...3.27)	<0.001
	APOBEC enr. score 4th Qu	726	1.48	(1.06...2.07)	0.020
	APOBEC2 >0.2	767	2.11	(1.48...3.01)	<0.001
	APOBEC3A >0.39		2.00	(1.43...2.81)	<0.001
	APOBEC3B >3.79		2.60	(1.93...3.51)	<0.001
	APOBEC3C >15.59		2.32	(1.70...3.17)	<0.001
	APOBEC3D >4.56		2.01	(1.42...2.86)	<0.001
	APOBEC3F >5.86		2.06	(1.41...3.02)	<0.001
	APOBEC3G >21.22		1.96	(1.41...2.74)	<0.001
	APOBEC3H >0.96		1.97	(1.44...2.70)	<0.001

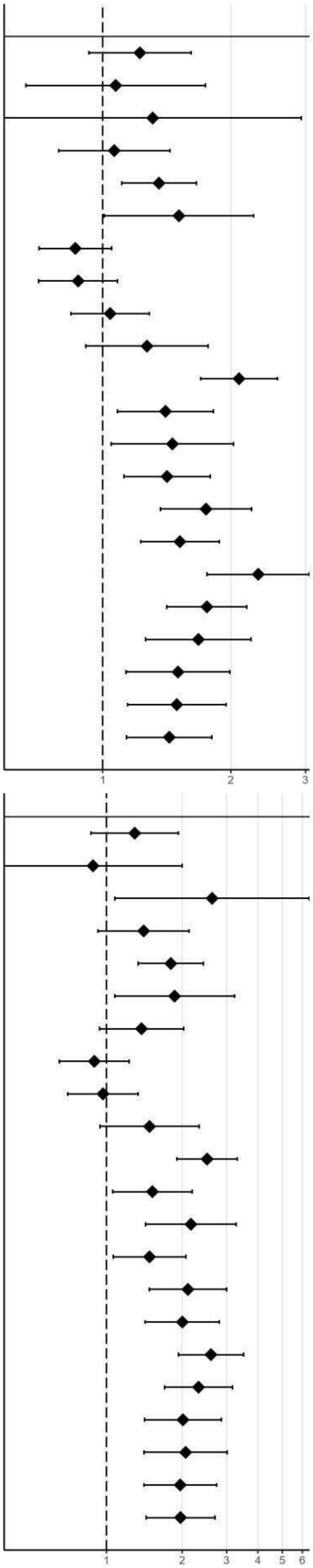

Figure S2.

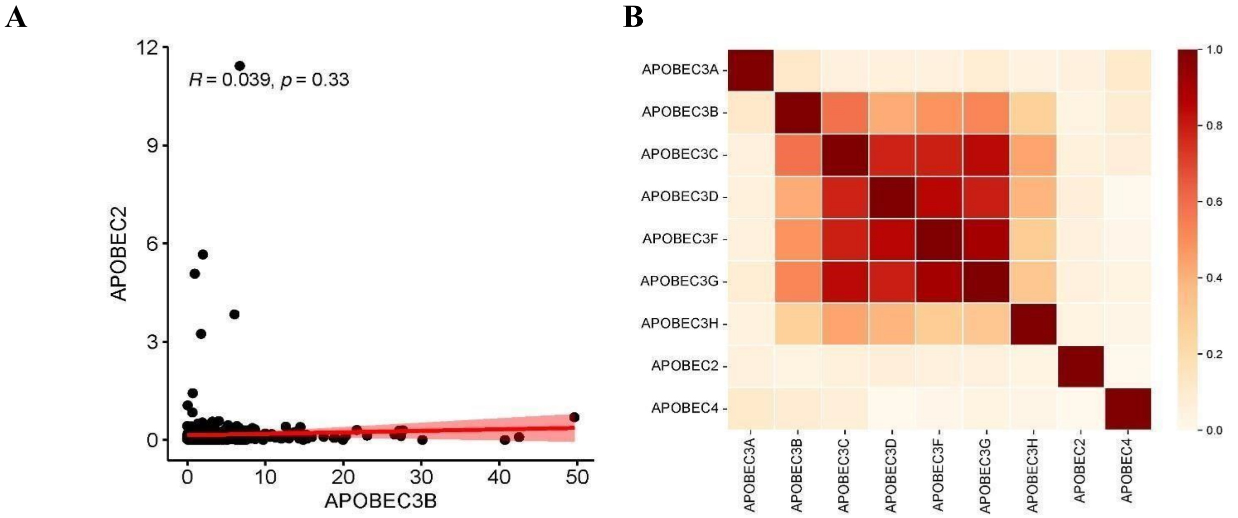
A
B
R = 0.039, p = 0.33
APOBEC2
APOBEC3B
APOBEC3A
APOBEC3C
APOBEC3D
APOBEC3F
APOBEC3G
APOBEC3H
APOBEC4

Figure S3.

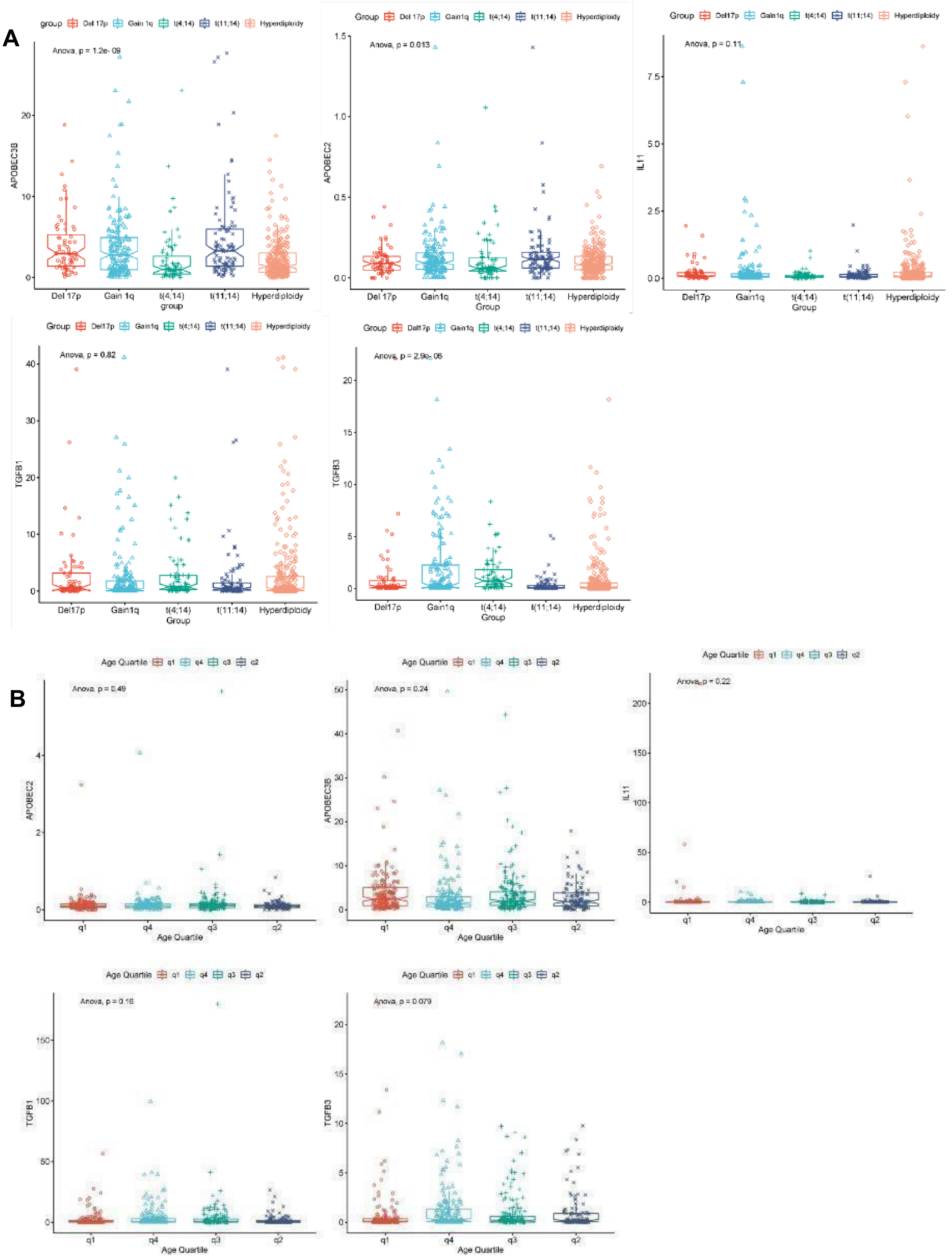
A
B
Anova, p = 1.2e-08
Anova, p = 0.013
Anova, p = 0.11
Anova, p = 0.82
Anova, p = 2.9e-06
Anova, p = 0.49
Anova, p = 0.24
Anova, p = 0.22
Anova, p = 0.16
Anova, p = 0.079
APOBEC3B
APOBEC2
IL11
TGFB1
TGFB3
Del 17p
Gain 1q
t(4;14)
t(11;14)
Hyperdiploidy
Group
Age Quartile
q1
q4
q3
q2

Figure S4.

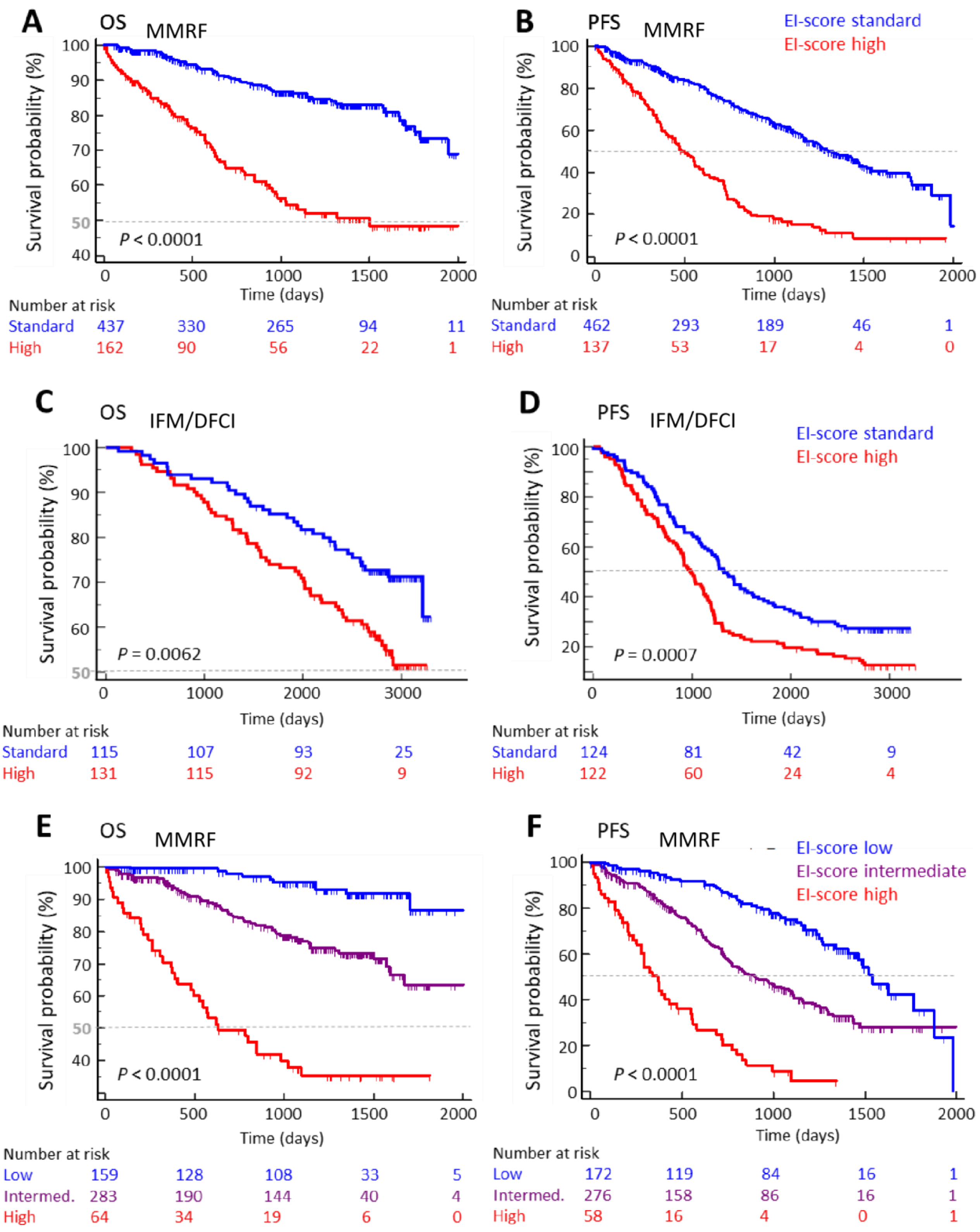
A
OS MMRF
Survival probability (%)
P < 0.0001
Time (days)
Number at risk
Standard 437 330 265 94 11
High 162 90 56 22 1
B
PFS MMRF
EI-score standard
EI-score high
Survival probability (%)
P < 0.0001
Time (days)
Number at risk
Standard 462 293 189 46 1
High 137 53 17 4 0
C
OS IFM/DFCI
Survival probability (%)
P = 0.0062
Time (days)
Number at risk
Standard 115 107 93 25
High 131 115 92 9
D
PFS IFM/DFCI
EI-score standard
EI-score high
Survival probability (%)
P = 0.0007
Time (days)
Number at risk
Standard 124 81 42 9
High 122 60 24 4
E
OS MMRF
Survival probability (%)
P < 0.0001
Time (days)
Number at risk
Low 159 128 108 33 5
Intermed. 283 190 144 40 4
High 64 34 19 6 0
F
PFS MMRF
EI-score low
EI-score intermediate
EI-score high
Survival probability (%)
P < 0.0001
Time (days)
Number at risk
Low 172 119 84 16 1
Intermed. 276 158 86 16 1
High 58 16 4 0 1

Figure S5.

A **All MMRF patients**

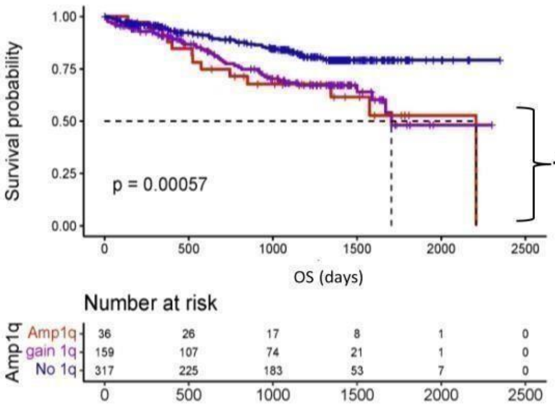

EI-score applied to Gain/Amp 1q

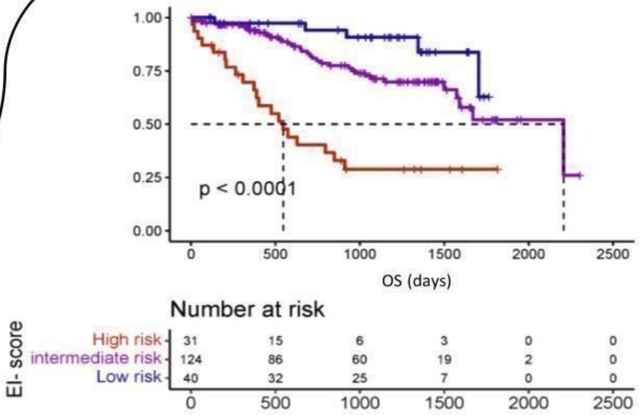

EI-score applied only to patients with +1q	% patients
**Low**	20% amp 1q<br>80% gain 1q
**Intermediate**	16% amp 1q<br>84% gain 1q
**High**	26% amp 1q<br>74% gain 1q

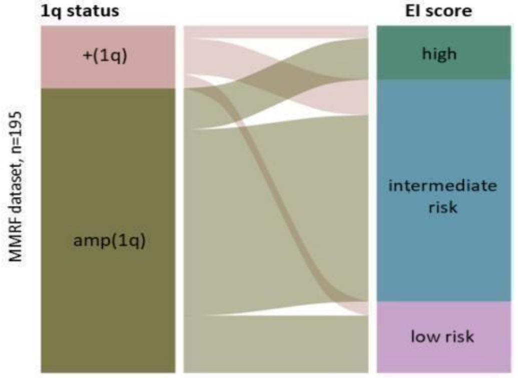

B **All MMRF patients**

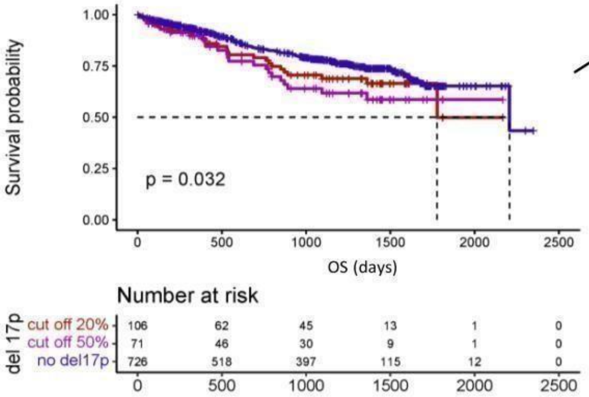

EI-score applied to del(17p), cut-off=20%

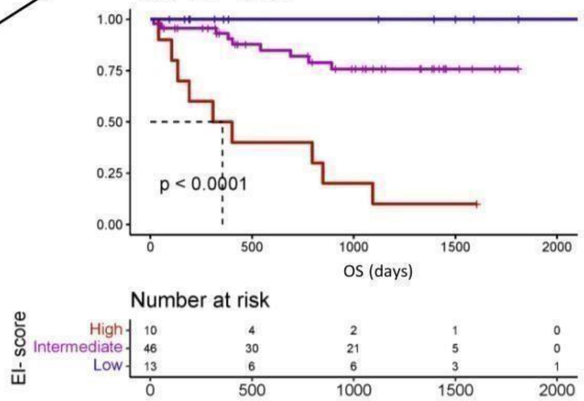

EI-score appl. to del17p >20%	%patients w CCF del(17p) >50%	%patients w CCF del(17p) <20% & >50%	%patients with *TP53* mut
**Low**	70%	30%	0%
**Intermediate**	60%	40%	26%
**High**	90%	10%	40%

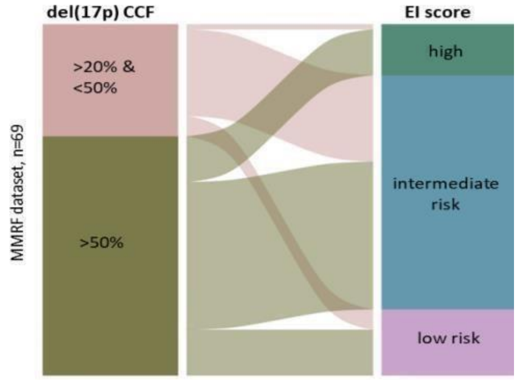

Figure S6.

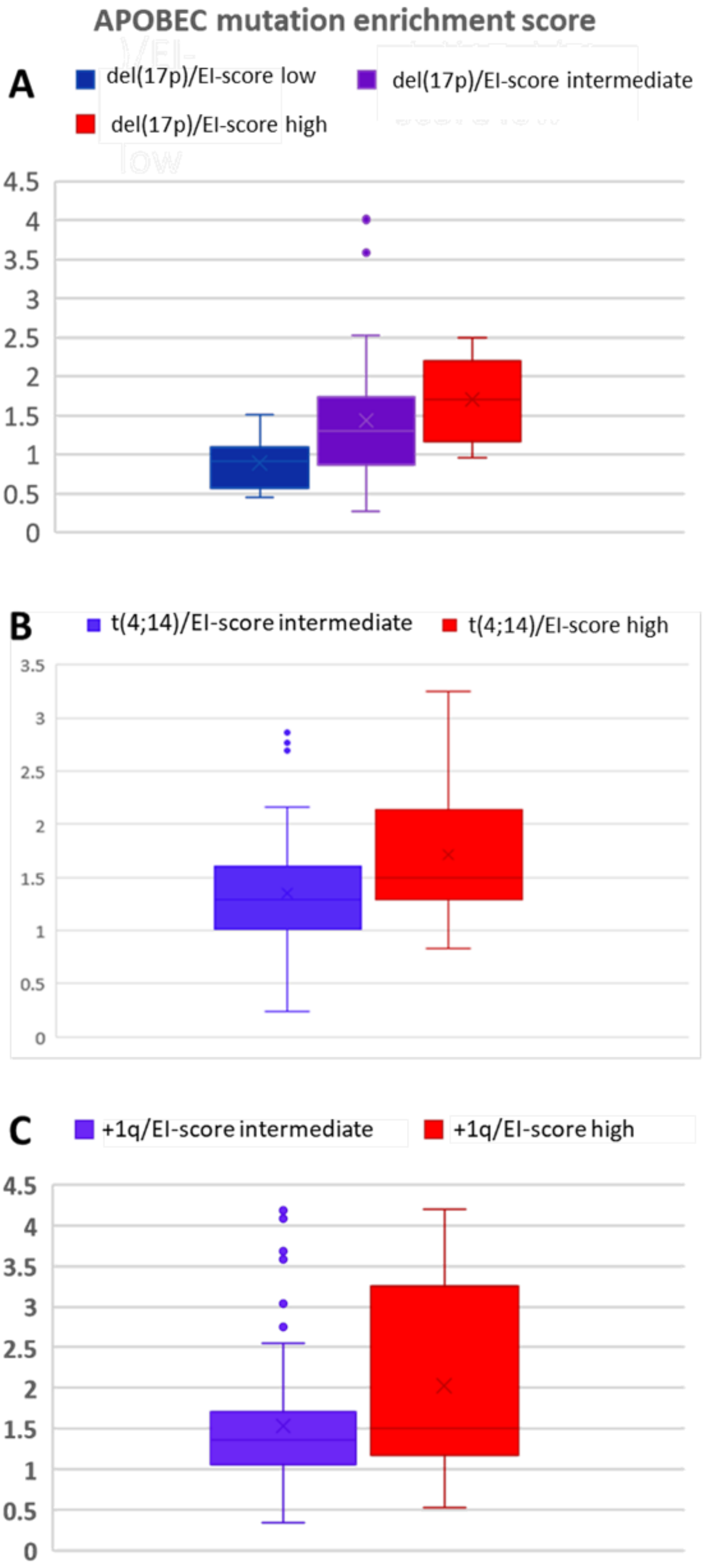
APOBEC mutation enrichment score
A
del(17p)/EI-score low
del(17p)/EI-score intermediate
del(17p)/EI-score high
4.5
4
3.5
3
2.5
2
1.5
1
0.5
0
B
t(4;14)/EI-score intermediate
t(4;14)/EI-score high
3.5
3
2.5
2
1.5
1
0.5
0
C
+1q/EI-score intermediate
+1q/EI-score high
4.5
4
3.5
3
2.5
2
1.5
1
0.5
0

Figure S7.

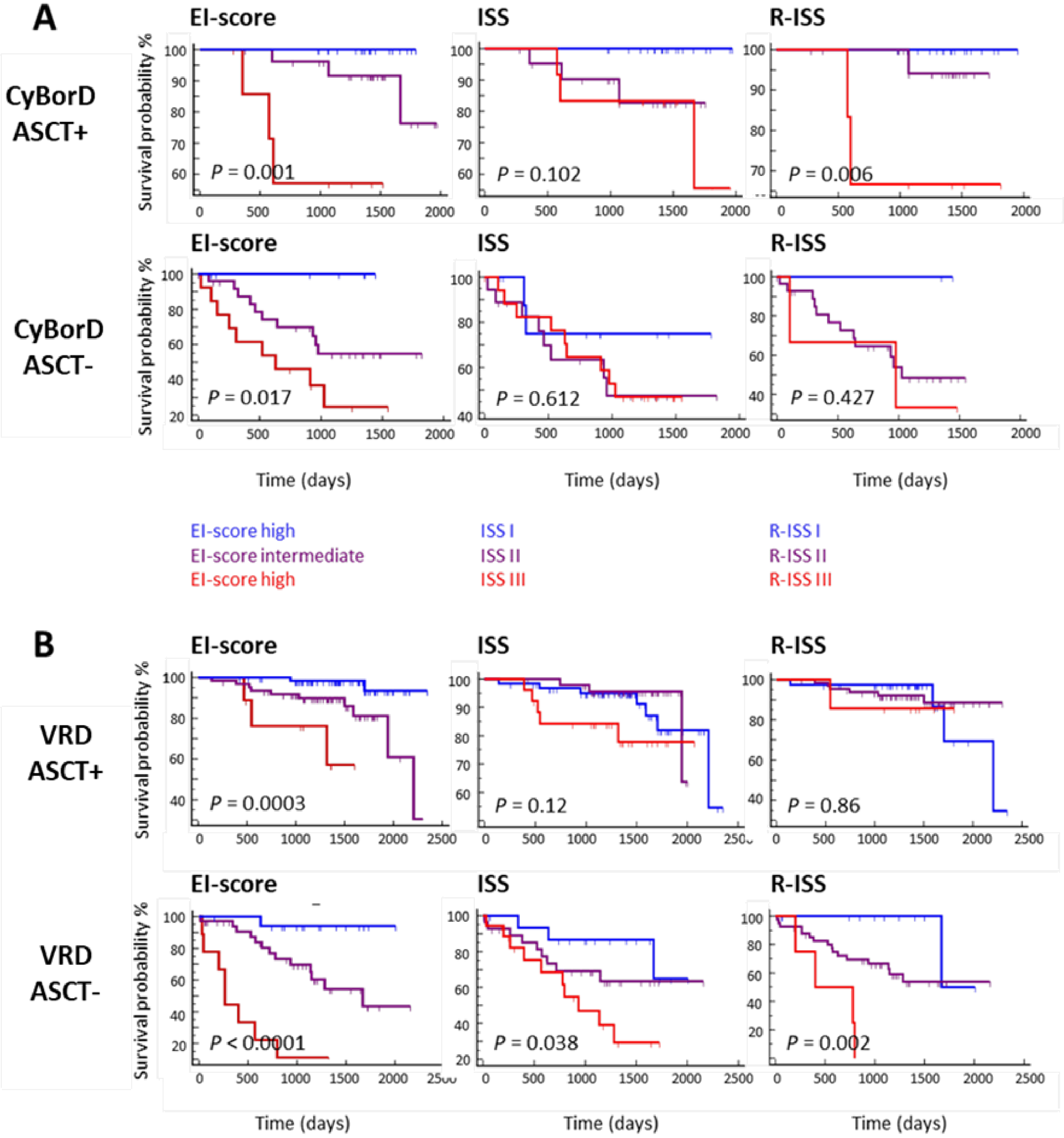

A
B
CyBorD
ASCT+
CyBorD
ASCT-
VRD
ASCT+
VRD
ASCT-
EI-score
ISS
R-ISS
Survival probability %
Time (days)
P = 0.001
P = 0.102
P = 0.006
P = 0.017
P = 0.612
P = 0.427
P = 0.0003
P = 0.12
P = 0.86
P < 0.0001
P = 0.038
P = 0.002
EI-score high
EI-score intermediate
EI-score high
ISS I
ISS II
ISS III
R-ISS I
R-ISS II
R-ISS III

Figure S8.

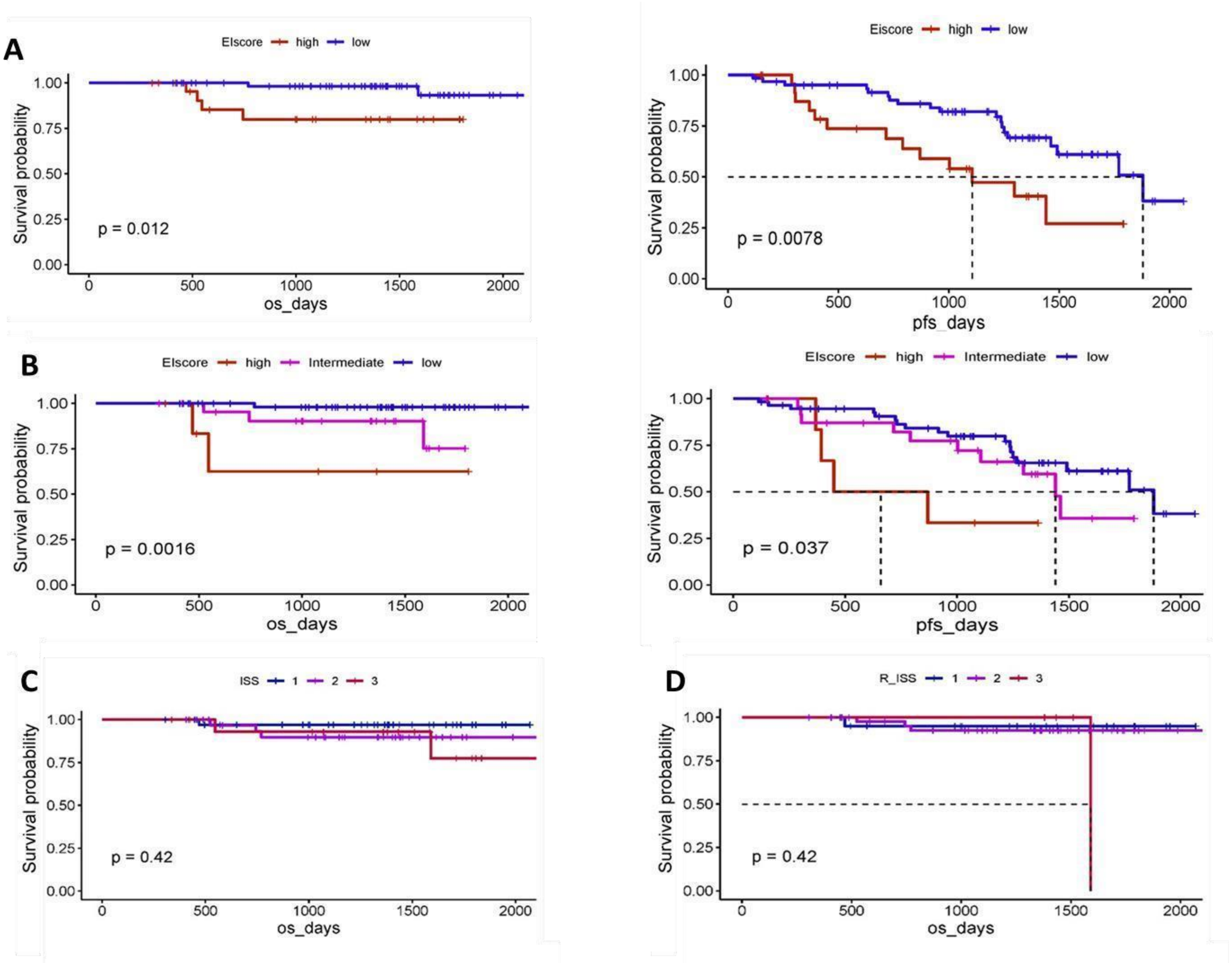
A
B
C
D
EIscore high low
Eiscore high low
EIscore high Intermediate low
ISS 1 2 3
R_ISS 1 2 3
Survival probability
os_days
pfs_days
p = 0.012
p = 0.0078
p = 0.0016
p = 0.037
p = 0.42
p = 0.42

Figure S9.

**Table S1.** Summary of patient baseline characteristics.		**MMRF CoMMpass study**	**IFM/DFCI 2009 study**
**No. of total patients**		1151 (1143 with survival data, 772 with RNA seq data, 725 with WGS/WES)	263 with survival and RNA seq data
**Gender (%)**	**Male**	60%	60%
	**Female**	40%	40%
**Age at diagnosis (years)**	**Mean**	62.91	56.5
	**Median (Q1-Q3)**	63 (56-70)	59 (53-62)
**PFS (days)**	**Mean**	733	1383.69
	**Median (Q1-Q3)**	628 (256.50-1152)	1157 (651.2-2099.7)
**OS (days)**	**Mean**	922	2405.33
	**Median (Q1-Q3)**	1066 (408- 1444)	2773 (1988.5-2948.5)
**ISS disease stage**	**I**	39%	31%
	**II**	37%	49%
	**III**	24%	20%
**R-ISS disease stage**	**I**	26%	14%
	**II**	64%	72%
	**III**	10%	14%
**Aberrant Cytogenetic detection method**		SeqFISH	FISH
**Aberrant cytogenetics**	**t(4;14)-WHSC1**	13%	11%
	**t(11;14)- CCND1**	20%	-
	**t(14;16)- MAF**	4%	11%
	**+1q**	38% (cut off 20%)	36% (cut off 30%)
	**Amp 1q**	8% (cut off 20%)	-
	**del(17p)**	13% (cut off 20%)	12% (cut off 9%)
	**Hyperdiploidy**	58%	-

Table S2. Incorporation of EI-score gene expression information improves the performance of established risk classifiers in the IFM/DFCI 2009 dataset.

Multivariate model	Cox Proportional Hazard Regression				ML Model ROC-AUC			n	
**Progression free survival (PFS)**	**Likelihood ratio**	**Wald test**	**Log rank test**	**Concordance index (C_i)**	**Random forest**	**gradient boosting**	**Negative binomial**	**total**	**events**
**1) ISS**	**4.47**	**4.51**	**4.53**	**0.549**	**0.5**	**0.5**	**0.5**	260	207
ISS + *APOBEC2, APOBEC3B*	13.21	13.82	13.96	0.582	0.54	0.57	0.58	260	207
ISS + *IL11, TGFB1, TGFB3*	14.75	14.92	15.04	0.581	0.54	0.59	0.57	260	207
ISS + APOBECs + Cytokines	21.88	22.53	22.81	0.603	0.51	0.62	0.63	260	207
**2) R-ISS**	**4.93**	**4.93**	**4.92**	**0.544**	**0.53**	**0.52**	**0.51**	254	203
R-ISS + *APOBEC2, APOBEC3B*	16.66	17.45	17.59	0.585	0.59	0.6	0.57	254	203
R-ISS + *IL11, TGFB1, TGFB3*	16.17	16.19	16.35	0.581	0.56	0.62	0.56	254	203
R-ISS + APOBECs + Cytokines	26.27	27.12	27.41	0.603	0.55	0.62	0.64	254	203
**3) R2-ISS**	**12.98**	**13.04**	**13.1**	**0.585**	**0.52**	**0.57**	**0.55**	192	154
R2-ISS + *APOBEC2, APOBEC3B*	32.25	33.28	34.17	0.636	0.53	0.63	0.65	192	154
R2-ISS + *IL11, TGFB1, TGFB3*	20.22	19.84	20.1	0.602	0.5	0.59	0.57	192	154
**R2-ISS + APOBECs + Cytokines**	**37.1**	**38.29**	**39.22**	**0.643**	**0.54**	**0.65**	**0.65**	192	154
**4) blood parameters (ß2M, LDH)**	**3.29**	**3.3**	**3.31**	**0.551**	**0.57**	**0.57**	**0.54**	247	196
ß2M + LDH + *APOBEC2, APOBEC3B*	12.27	12.78	12.92	0.579	0.54	0.57	0.55	247	196
ß2M + LDH + *IL11, TGFB1, TGFB3*	16	16.43	16.62	0.58	0.56	0.61	0.59	247	196
**ß2M/LDH + APOBECs + Cytokines (EI-score)**	**23.48**	**24.43**	**24.77**	**0.601**	**0.56**	**0.54**	**0.55**	247	196
**5) gene expression only:**	-	-	-	-	-	-	-	-	-
APOBEC2, APOBEC3B	9.18	9.79	9.92	0.557	0.48	0.55	0.55	266	212
IL11, TGFB1, TGFB3	12.27	12.25	12.38	0.567	0.49	0.58	0.57	266	212
APOBECs + Cytokines	19.54	19.96	20.29	0.587	0.53	0.63	0.61	266	212
**Overall survival (OS)**	**Likelihood ratio**	**Wald test**	**Log rank test**	**Concordance index (C_i)**	**Random forest**	**gradient boosting**	**Negative binomial**	**total**	**events**
**1) ISS**	**8.15**	**8.17**	**8.29**	**0.577**	**0.57**	**0.56**	**0.55**	259	98
ISS + *APOBEC2, APOBEC3B*	16.2	16.84	17.39	0.623	0.6	0.6	0.62	259	98
ISS + *IL11, TGFB1, TGFB3*	35.2	36.46	37.32	0.65	0.67	0.67	0.68	259	98
ISS + APOBECs + Cytokines	42.27	42.77	44.59	0.665	0.69	0.69	0.68	259	98
**2) R-ISS**	**7.43**	**7.47**	**7.43**	**0.563**	**0.55**	**0.54**	**0.53**	253	97
R-ISS + *APOBEC2, APOBEC3B*	17.02	17.8	18.18	0.62	0.6	0.61	0.61	253	97
R-ISS + *IL11, TGFB1, TGFB3*	33.92	35.07	36.01	0.644	0.63	0.68	0.66	253	97
R-ISS + APOBECs + Cytokines	42.35	43.2	44.75	0.668	0.66	0.68	0.67	253	97
**3) R2-ISS**	**29.12**	**29.71**	**30.04**	**0.672**	**0.64**	**0.68**	**0.66**	191	72
R2-ISS + *APOBEC2, APOBEC3B*	40.08	40.34	41.88	0.706	0.67	0.7	0.69	191	72
R2-ISS + *IL11, TGFB1, TGFB3*	50.14	49.72	52.24	0.709	0.7	0.72	0.71	191	72
**R2-ISS + APOBECs + Cytokines**	**60.38**	**57.24**	**62.42**	**0.728**	**0.7**	**0.73**	**0.74**	191	72
**4) blood parameters (ß2M, LDH)**	**7.5**	**7.42**	**7.53**	**0.583**	**0.55**	**0.57**	**0.58**	246	92
ß2M + LDH + *APOBEC2, APOBEC3B*	13.99	14.23	14.68	0.622	0.63	0.61	0.62	246	92
ß2M + LDH + *IL11, TGFB1, TGFB3*	36.28	37.57	38.58	0.647	0.66	0.68	0.66	246	92
**ß2M/LDH + APOBECs + Cytokines (EI-score)**	**42.81**	**42.65**	**44.53**	**0.663**	**0.71**	**0.69**	**0.67**	246	92
**5) gene expression only:**	-	-	-	-	-	-	-	-	-
APOBEC2, APOBEC3B	9.89	10.87	11.21	0.59	0.67	0.67	0.67	265	100
IL11, TGFB1, TGFB3	27.41	28.68	29.34	0.622	0.65	0.64	0.63	265	100
APOBECs + Cytokines	36.06	36.82	38.37	0.647	0.65	0.65	0.66	265	100
